# Clinical Experiences in Private Practice—The Curative Relation of Vaccination to Whooping-Cough, with Clinical Cases

**Published:** 1870-12

**Authors:** Z. C. McElroy

**Affiliations:** Zanesville, Ohio


ARTICLE XXXIX.

### CLINICAL EXPERIENCES IN PRIVATE PRACTICE—THE CURATIVE RELATION OF VACCINATION TO WHOOPING-COUGH, WITH CLINICAL CASES.

REPORT OF COMMITTEE ON NEW REMEDIES TO THE MUSKINGUM COUNTY MEDICAL SOCIETY, AT NOVEMBER SESSION, 1870.

By Z. C. McELROY, M.D., Zanesville, Ohio.

No special novelty in the therapeutical world has transpired during the past month, though the demand for new remedies by the profession has by no means fallen off. The career run by different new remedies is as diversified as that of different men, having an apparently equal start in the race for life. Some require many years to get even a recognition in pharmacopias; while others, as chloral, fill the world of mind in both hemispheres of the globe, in a few days. The career of chloral has been as remarkable as that of Protoplasm. *The Methodist Quarterly Review* for October, says: "It is a signal proof of the brilliant genius and eminent standing of Prof. Huxley, that in one fortnight he was able to install the term 'Protoplasm' among the key words of the English language, and to fill the higher mind of the world with excitement at its alarming import." The belief in the celestial origin of man, at least among animals, had, up to a few years since, never been questioned by any man of science, having sufficient standing to command, even for a single moment, a casual readers attention outside of his own neighborhood. Hundreds of thousands of the best cultivated intellects of the civilized world have not only read, but pondered, Huxley's remarkable lecture on "Protoplasm, or Physical Basis of Life," published in London, only eighteen months since, and delivered to an Edinburgh evening audience less than two years ago! Life the result of physics! What a startling announcement? And how natural that the cultivated mind of the world should be filled with excitement at its alarming import. The theological mind felt that the very foundations of religious belief were again threatened by science. The higher medical mind was not slow to see that it was the handwriting on the wall foreshadowing still greater changes in medicine as now taught the world even in schools, and practised, alike by regulars and irregulars. Changes so important are very properly interpreted as meaning that men must speedily occupy the upper seats in the world's great medical Sanhedrim. Reputations which were believed to be world-wide, and secure beyond any contingency, it was clearly seen must undergo immense shrinkage and modification.

The effect is already beginning to be seen in medical periodical litenature. The paragoric-good-for-a-child's-belly-ache-style of reports of cases are occasionally relieved by those reporting temperature, circulation, and respiration, to the exclusion of vague speculations concerning the vital force. Time will not be long in disclosing whether the profession will fall into line with the world's progress, or cling to the *status quo* till a great schism thunders its lesson in their ears.

My purpose this month is to invite your attention to the curative, or remedial relations existing between vaccination and whooping-cough.

That "vaccination is good for the whooping-cough," using recognized modes of announcing the curative properties of any agency, or measure, would not likely fall now upon your ears for the first time.

Before any of us began to practice, or even study our professions, it was lauded as a specific, tried in a Paris hospital, failed, and was dropped out of medical literature; for vaccination is not even mentioned in the most recent works on general practice, or the special management of the diseases of childhood, as a proper remedial measure in the professional management of Pertussis. In one of those just superceded by later authors, "Churchill's Diseases of Children," the American editor, Dr. Keating, of Philadelphia, says, in a note: "I have tried vaccination in several cases, (of whooping-cough), and have every reason to be satisfied with the result, having found it to modify both the severity and length of the affection."

This, it appears to me, is a correct statement of the present condition of professional judgment in regard to vaccination as a remedial measure for whooping-cough. Tried, succeeded sometimes, and in some hands, and, perhaps it ought to be added, under some circumstances. In other hands, and under different circumstances, it has apparently failed, and stands now as one of those dernier resorts when all other things have failed.

What is whooping-cough? With the anterior phenomena, and some of the interior disclosed by autopsies, most of us, are, perhaps, familiar.

Medical authors of the first half of the present century were fruitful in hypotheses, speculations, and conjectures to account for the phenomena. Spasm of the air-passages had a formidable list of supporters. Inflammation of the lining membrane of the air-passages had hardly a less numerical array of influential advocates. Then a third class mixed spasm and inflammation together, and claimed that as the only hypothesis which would account for the phenomena. A somewhat older class of medical authorities referred it to irritation of the stomach and lungs. By Sydenham and his cotemporaries it was ascribed to a physical or chemical irritant, introduced from without into the larynx; or generated within, and produced its peculiar effects on its way to the outside through the breath. Various were the shades of difference between the pathologists of the last two centuries, some referring it to a miasm, others to minute insects, etc., etc.

The very latest authorities of our own times dispel these conjectures, hypotheses, and speculations, and simply state it to be due to a specific poison, and place it in the same class as small-pox, measles, scarlatina, etc.; contagious, and infectious, and communicable from one human being to another; having a definite course to run, like them, ending in apparently complete, or incomplete recovery, and sometimes in death.

Discussions, based on the exterior phenomena presented by the human body, whether physiological or pathological, it is thus seen, might go on forever without arriving at conclusions which would satisfy any considerable number of men, for any length of time, even if they should escape being toppled over by some advance of science. The conjectures, speculations, or hypotheses of to-day, will in their time give place to the products of some exhuberant immagination to-morrow, and so on indefinitely forever.

Supposing that modern molecular science, which traces the combinations of atomic particles of inorganic matter through various stages of complexity, up to molecular forms of organic structures, is the ultimate simplicity to which science can carry us in the study of organic life, it is very interesting to note, in the study of the varying ideals of the pathology of whooping-cough through two centuries only, how near Sydenham came to the naked scientific reality, having just missed it by regarding it as a local disease. Combining the pathology of Sydenham with that of Aitkin, and the result accounts, scientifically, for all its phenomena. The idea of the local nature of disease is now the greatest barrier in the way of progression to scientific pathology.

Molecular physics, rejecting all hypotheses, speculations, and conjectures concerning the human body, seeks explanations of its varied phenomena in the molecular forms of its varied structures and viscera; tracing inorganic matter through its various chemical transformations, from the inorganic world, up to organic forms of structure, in which it finds conserved the energy evolving all its phenomena, mechanical, chemical, thermal, emotional, sensory, intellectual, and physiological. In ever narrowing circles, the added facts of life, or the facts of the physical organization of the human body, have at length identified its physical and psychological unities.

These are, 1st, inorganic material; 2d, organic forms; 3d, motion, or chemical changes in the molecular forms of structure. In material, the human body is composed of certain inorganic elements, definite alike in number and relative proportion. These definite materials, by the earths forces, are worked into, first, vegetable organic forms, which, as the starting point of animal life, are by the same forces worked into definite molecular forms of structure for each function evolved. Function is therefore, the molecular forms of structure speaking, and depends on changes of matter.

In the pathological state of so-called whooping-cough, is function changed? Most assuredly, as the cough, the wasting, loss of molecular forms of structure, in the lungs and brain, and death, as a too frequent result, clearly demonstrates.

Going down to the bottom of simplicity in reference to chemical and mechanical effects on a physiological human body, it is found only possible to do three things by any means, or mode of force whatever. These are, to accellerate or retard motion, or chemical changes, and change organic forms.

In reducing the phenomena of whooping-cough to ultimate simplicities, or unities, it seems to me this statement covers all the symptoms. Motion in the interest of repair retarded; motion in the interest of waste accelerated; and the molecular forms of structure undergoing changes. It is, then, by modern pathologists, very properly classed among the so-called exanthemetha as small-pox, measles, scarlatina, vaccina, syphillis, etc., and differs from either of them only in the extent and velocity with which its mode of force interferes with normal forms and motion.

The cotemporaries of Sydenham had a nearer conception of the cause, and the *modus operandi* of virus, or form-changing mode of force, on which whooping-cough depends, than any pathologists down to the present time, *viz.*, a physical or chemical irritant—and if there is any conception with which the term irritation is coupled, it is to disturb both motion and form.

The duration of whooping-cough, that is, the length of time requisite for it to complete its form-changing work in any given human body, is by no means fixed; for who can assign to it limits such as he could, with the utmost confidence, to measles, cow-pock, scarlatina, or small-pox? On the contrary, its motion is so slow, and interfered with by so many physical contingencies, as the condition of the earth's force, age, etc., that no one feels safe to assign limits, except vaguely, to its probable duration in any given case.

The list of remedial agencies recommended by famous authorities during the last century is very large, and includes some with very opposite properties. Those employed commonly had some correspondence with the hypothetical views of its pathology. Then, those who regarded it as inflammatory, made use of antiphlogistics, bleeding, calomel, and antimony. Those who regarded it as spasmodic, prescribed mainly the so-called anti-spasmodics. For the mixed pathology, these remedial agencies were commonly combined. The means pointed out by the most modern authorities are, for the most part, hygienic, using mild eliminators, and those remedial agents which retard motion in the interest of waste, commencing with chloral, taking in opium and belladona, and ending with camphor. While the eliminants commence with emetics, and wind up with calomel, blisters, and cantharides. The gaps in such plans of treatment are occasionally filled up with agents accelerating repair, as nitric acid, oxides of zinc and arsenic. Lastly, all these failing, change of air,—more ozone to burn up dead tissue and quicken motion; or the ammoniacal atmosphere of gas works, which does about the same thing.

Aitken, a very late English authorithy, states "that small-pox and whooping-cough may co-exist in the same person; and a very common and fatal combination is measles and whooping-cough. Whooping-cough," he goes on to say, "and cow-pock are not unfrequently combined. Indeed, the lower classes erroneously look upon vaccination as in many instances a cure for whooping-cough."

It was " common people" who believed, because they knew from experience, the court of last resort in the profession, that cow-pock protected them from small-pox, and just such men then, as Aitken is now, fought poor Jenner for twenty years, because he had experimented with cow-pock, and demonstrated that the belief of the common people was right, and, therefore, urged vaccination on professional attention. He was hounded out of all medical societies, and almost out of human society, for his alleged heresy. But Jenner still lives, which can hardly be said of any of his persecutors.

Though vaccination has been fully acquiesced in as a complete protection against variola for half a century, doubts of its efficacy and propriety have recently arisen in the public and professional mind, and it is again up for discussion and settlement at the bar of public and professional opinion. No result has, as yet, been reached, but it is clear that vaccination has materially declined in public estimation within a few years.

But the power of vaccination, under some circumstances, to protect against small-pox is as well established as the law of gravity. Vaccination is a smaller evil, accepted to protect against a greater.

Every mode of force, as the virus of insects and serpents, small-pox, scarlet-fever, measles, vaccination, and in this category, the mode of force producing the phenomena called whooping-cough must be placed, produce these effects in the human body by changing the molecular forms of structures, the duration of the phenomena depending on the velocity of motion by which this is accomplished, as measured partly by the heat evolved as shown by the thermometer.

And, among their results in the human body, it appears probable, will be found explanations of the so-called idiosyncracies, or individual peculiarities, as well as some peculiarities of mind and morals, manifested later in life as well,—that is to say that the peculiarities of body, as well as of mind, observed in some human beings, before, as well as during adult life, will ultimately be found due to changes of molecular forms of structure by these eruptive and non-eruptive form-changing diseases, some of which, as whooping-cough, are mainly passed through during child-life.

Aside from the results of empirical experience in regard to the so-called curative power of cow-pock over whooping-cough, that result could be predicted by philosophy and molecular science, just as chloral was worked out in the mind of Deibriech, in advance of empirical experiment. Whooping-cough is due to a mode of force whose velocity is very slow, extending over from one and one-half to several months; while cow-pock has a velocity very uniform at all stages of life.

That it should be able to communicate a higher velocity, in fact its own velocity, to whooping-cough, is altogether probable, aside from its repeated demonstration by experience.

Guided by these general principles, it seems not improbable that most of these formidable form-changing modes of force may ultimately be so antagonistic as to materially modify their results in the human body. Prof. Boeck's proposal to treat syphilis by repeated inoculations by the syphilitic virus is strictly philosophical, because, in that way, he communicates a higher velocity to the changes, and secures, in a few months, results which should otherwise be spread over, in most cases, the remainder of life in each individual case. Had he turned his attention to other organic agencies increasing the velocity of motion of syphilis, much greater results might have been reached. This seems all the more probable, as all the recognized successful remedial measures for syphilis have for their end simply increase of motion, both in the interest of repair and waste.

The following clinical cases, treated during the present year, may serve to show that the motion of cow-pock can be successfully employed to increase the motion of whooping-cough, terminating both simultaneously.

The family of Mr. D. H. K., market-gardener, living five miles down the river, in common with many other families, both in the city and country, contracted whooping-cough early in the summer. It seems to me there were six of the family suffering from it at the same time. The youngest child, a year old, perhaps, became very sick, so much so as to impress the parents with doubts of his recovery. Without seeing it, the emetic, purgative, and anodyne plan of management, so-called, had been prescribed for several weeks. He became so seriously ill that his parents thought it best for me to see him. He was found very unwell, breathing very rapid and short, pulse rapid and thready, and a range of temperature, by thermometer, much above natural, though not recollected, precisely; think it was 105½. I gave him 15 drops tinct. ver. veride, and, as no effect was manifest in half an hour, a like dose was repeated. This was given to check motion and procure prompt elimination from his lungs, as well as stomach. The sickness and vomiting lasted about an hour and a half, during which his head was kept very low, so as to facilitate, by gravity, the elimination of the matter with which the lungs seemed materially loaded. The improvement in his breathing and pulse was very marked at the close of vomiting, so that he soon dropped off in a sound sleep. No other treatment was prescribed that day, but a dose of castor-oil when he woke up and nursed. In three days, I went down, expressly to vaccinate Mr. K.'s family. Three of the older children had been vaccinated several years previously. But all suffering from whooping-cough were inoculated; and, strange to say, all had sore arms in due time, those previously vaccinated running through the stages in about a week, while in the remainder the usual two weeks were occupied in getting through with it. When they had recovered from the vaccination, the whooping-cough had disappeared. By a previous arrangement, the children of Mr. J. D., living two miles from

Mr. K., were to meet me there, to be vaccinated for the same purpose, but did not get there until after I had left. Two of them were brought to my office a few days after, and were vaccinated. At a proper time, one of the pocks was ruptured with a common sewing-needle, by the mother, and inserted in the arms of the remaining members of the family. In reporting to me afterwards, the mother said she had no whooping-cough after the children recovered from the vaccination; that they were all very sick, and she thought I had used very good matter.

Several of her neighbors obtained crusts from her, and used them, and, in all, the whooping-cough and vaccination disappeared together. Quite a number of other cases, isolated, were managed in the same way, and with the same results. This is only the present year's experience, and there were no failures.

In past years cases were so managed, but not so intelligently, and, so far as memory serves, with hardly the same uniform success.

I conclude, therefore, as well from the results of experience, as philosophical considerations, that in cow-pock we have the means of hurrying a majority, at least, of cases of whooping-cough through its stages within the two weeks occupied by the former; and it is worth while to consider whether vaccination should not always be delayed for the purpose, unless imperatively demanded by other considerations.

And there may be some conditions needful to obtain a large average success, which I have not identified, but which can doubtless be made out, if looked for by competent observers.

---

To Detect Uric Acid in the Blood.—Dr. Garrod's ingenious plan of detecting uric acid, which is always in great excess in gout, is as follows: It consists in obtaining the crystals of uric acid on a thread placed in a mixture of the serum of the blood, or of the fluid from a blister, with acetic acid, in the proportions of six minims of the acid to each fluid drachm of the serum.—*Richmond and Louisville Medical Journal.*

