# Errors

**Published:** 1871-03

**Authors:** 


ERRORS.—Dr. McElroy, of Zanesville, Ohio, writes to us a letter, pointing out the formidable list of *eighteen* typographical errors in his article published in the December number of the EXAMINER. A careful examination of the original manuscript in the hands of the printers shows, that six of the errors claimed exist in the manuscript; nine others are so slight as not materially to alter the meaning of the writer; and the remaining three are as follows: Page 736, 29th line from the top, for *anterior* read *exterior;* page 741, 16th line from the top, for *Deilriech* read *Liebriech;* page 741, 26th line from the top, for *anagonlistic* read *antagonized.*

